# Acylhydrazone Derivative A5 Promotes Neurogenesis by Up-Regulating Neurogenesis-Related Genes and Inhibiting Cell-Cycle Progression in Neural Stem/Progenitor Cells

**DOI:** 10.3390/molecules29143330

**Published:** 2024-07-16

**Authors:** Xiaoliang Xiang, Xia Jiang, Hongwei Lin, Meixing Yu, Liming Wu, Rong Zhou

**Affiliations:** 1 Hunan Provincial Higher Education Key Laboratory of Intensive Processing Research on Mountain Ecological Food, College of Biological and Food Engineering, Huaihua University, Huaihua 418008, China; jiangxia285333006@163.com (X.J.); stannio@163.com (L.W.); suduzhongyao1234@163.com (R.Z.); 2 College of Chemistry and Materials Engineering, Huaihua University, Huaihua 418008, China; linhongwei1968@163.com; 3 Guangzhou Women and Children’s Medical Center, Guangzhou Medical University, Guangzhou 510180, China; yumeix509@163.com

**Keywords:** acylhydrazone derivative, neural stem/progenitor cells, neurogenesis, cell cycle

## Abstract

Adult neurogenesis involves the generation of functional neurons from neural progenitor cells, which have the potential to complement and restore damaged neurons and neural circuits. Therefore, the development of drugs that stimulate neurogenesis represents a promising strategy in stem cell therapy and neural regeneration, greatly facilitating the reconstruction of neural circuits in cases of neurodegeneration and brain injury. Our study reveals that compound A5, previously designed and synthesized by our team, exhibits remarkable neuritogenic activities, effectively inducing neurogenesis in neural stem/progenitor cells (NSPCs). Subsequently, transcriptome analysis using high-throughput Illumina RNA-seq technology was performed to further elucidate the underlying molecular mechanisms by which Compound A5 promotes neurogenesis. Notably, comparative transcriptome analysis showed that the up-regulated genes were mainly associated with neurogenesis, and the down-regulated genes were mainly concerned with cell cycle progression. Furthermore, we confirmed that Compound A5 significantly affected the expression of transcription factors related to neurogenesis and cell cycle regulatory proteins. Collectively, these findings identify a new compound with neurogenic activity and may provide insights into drug discovery for neural repair and regeneration.

## 1. Introduction

Neural stem/progenitor cells (NSPCs) are multipotent stem cells capable of proliferating and self-renewing in the developing and adult central nervous system [1]. These cells possess the ability to differentiate into various neural lineages, such as neurons, astrocytes, and oligodendrocytes. The dentate gyrus is a region of the hippocampal formation in which NSPCs are present and exhibit neurogenesis throughout life [2]. Neurogenesis in the dentate gyrus of the hippocampus is thought to be critical for learning and memory function [3]. Therefore, neurogenesis of NSPCs in the adult central nervous system (CNS) plays an important role in the repair of CNS injury and the recovery of neurodegeneration and also becomes a potential therapeutic application for nerve injury and neurodegenerative diseases [4,5,6]. Recently, accumulating evidence has demonstrated that inducing neurogenesis of adult neural stem cells in vivo by drugs is an effective strategy for the treatment of nerve injury and neurodegenerative diseases [7,8,9]. Metformin, a commonly used diabetes drug, can promote neurogenesis in adult NSPCs and enhance spatial memory formation in mice [10]. In addition, other bioactive compounds, such as saffron [11], lavender [12], basilicum [13], and rosa [14], have been discovered for their potential neurogenesis-inducing effects.

We previously synthesized some novel acylhydrazone compounds containing the 1,2,4-triazole structure, Compound A5, which exhibits excellent neuritogenic activity toward the Neuro-2a cells [15]. Neuro-2a is a neuronal cell type and a suitable in vitro model for studying neural development. The sustained growth of neurite is generally regarded as a hallmark event in the differentiation of Neuro-2a cells. Therefore, we speculate that Compound A5 can induce rapid cellular changes in neuronal differentiation or influence NSPC neurogenesis. To verify our hypothesis, in this study, NSPCs were used to examine the effect of Compound A5 on neurogenesis. Indeed, as we speculated, compound A5 significantly affects neurogenesis, inducing the differentiation of NSPCs into neurons and astrocytes and promoting the morphological maturation of newborn neurons.

To gain a deeper understanding of the molecular mechanism of compound A5 on neurogenesis, we applied RNA sequencing to assess and compare gene expression patterns after Compound A5 treatment. The result revealed that genes related to neurogenesis were up-regulated, while the down-regulated genes were mainly related to cell cycle progression. Finally, we confirmed that Compound A5 promotes neurogenesis by up-regulating neurogenesis-related genes and inhibiting cell-cycle progression in NSPCs. Although the specific molecular target of Compound A5 remains to be elucidated, these findings provide a novel approach for drug design in treating nerve injury and neurogenerative diseases.

## 2. Results and Discussion

### 2.1. Compound A5 Promotes Neuronal Differentiation and Neurite Outgrowth of Neuro-2a Cells

**Figure 1:**
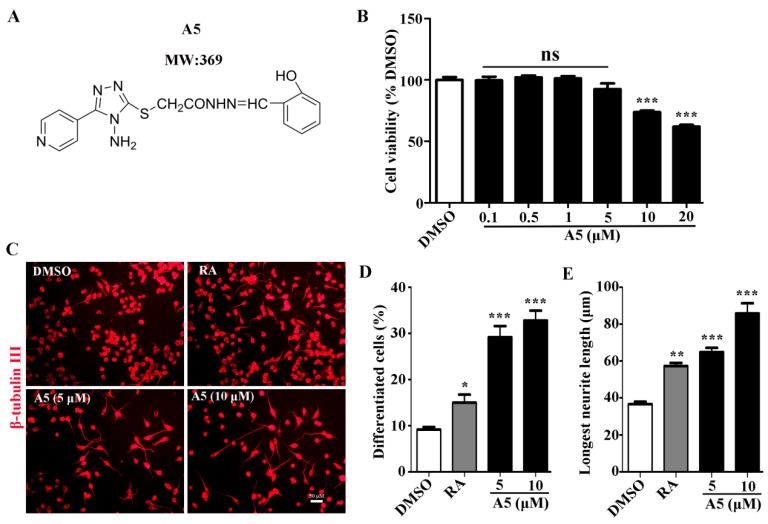
Compound A5 promotes differentiation and neurite outgrowth of Neuro-2a cells. (**A**) The chemical structure of Compound A5. (**B**) Neuro-2a cells were treated with Compound A5 in a concentration gradient (0.1, 0.5, 1, 5, 10, 20 μM) for 24 h. Cell viability was tested using an MTT assay. (**C**) Neuro-2a cells were treated with Compound A5 (5 and 10 μM) and RA (10 μM) and cultured under a differentiation medium for 48 h. Neurites were visualized using β-tubulin III antibody (red). Scale bar, 50 μm. The differentiation rate (**D**) and the longest neurite length (**E**) of each differentiated cell were calculated. One-way ANOVA followed by Tukey’s test. Error bars represent SEM (*n* = 3). ns, no significant differences; * *p* < 0.05, ** *p* < 0.01, *** *p* < 0.001.

Our previous findings demonstrated that the Compound A5 exhibits excellent neuritogenic activity in Neuro-2a cells. To explore the effects of Compound A5 on neuronal differentiation, Neuro-2a cells were exposed to various concentrations of Compound A5 (5 and 10 μM) (Figure 1A,B). Retinoic acid (RA, 10 μM) was used as a positive control. After 48 h of differentiation, cells were immunostained with an antibody against β-tubulin III to visualize the presence of neurite. As shown in Figure 1C, the untreated cells (DMSO) have a round shape with few neurites, and the RA-treated cells apparently display long neurites. Here, we compared the effects on the differentiation of Neuro-2a cells induced by Compound A5. Notably, in all aspects we examined, including differentiation rate (Figure 1D) and the longest neurite length (Figure 1E), Compound A5 exhibited stronger activities than RA did. Therefore, our results showed that Compound A5 promoted neuronal differentiation and neurite outgrowth of Neuro-2a cells in a concentration-dependent manner.

### 2.2. Compound A5 Enhances Neurogenesis in Cultured NSPCs

**Figure 2:**
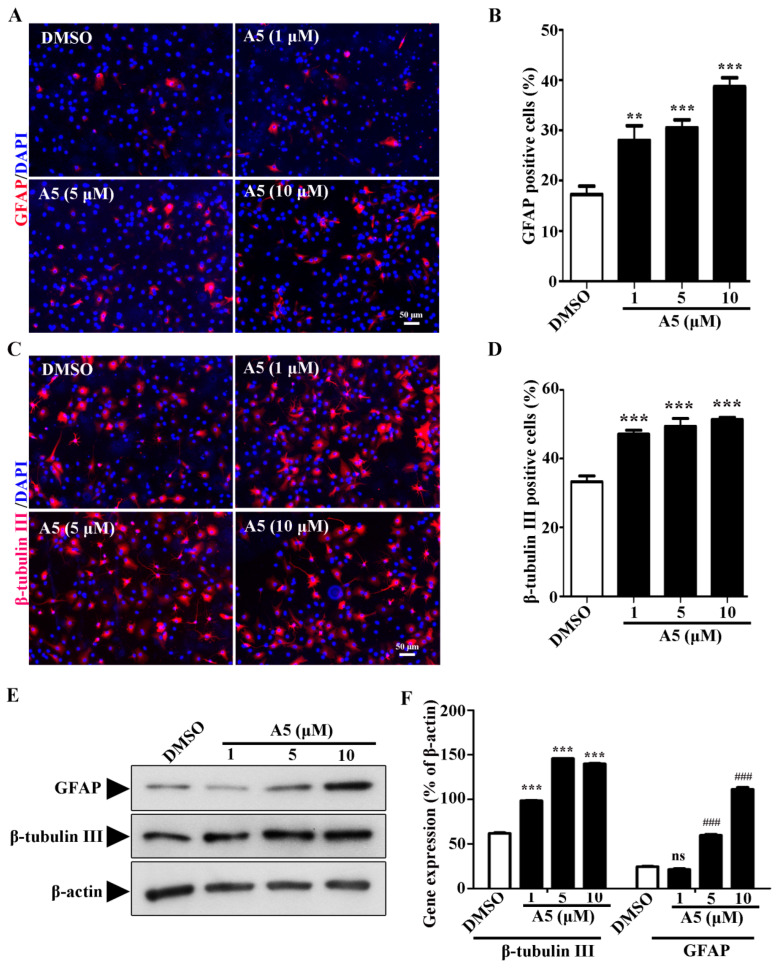
Compound A5 promotes NSPCs differentiation. NSPCs were differentiated for 5 days in the presence of DMSO or different concentrations of Compound A5. Immunostaining of astrocytes for GFAP (**A**,**B**), newborn neurons for β-tubulin III (**C**,**D**), and nuclei with DAPI. Scale bar, 50 μm. Quantification of β-tubulin III positive neurons and GFAP positive astrocytes differentiated from NSPCs. (**E**,**F**) Western blotting confirmed the presence of β-tubulin III and GFAP protein in treated and untreated cells. One-way ANOVA followed by Tukey’s test. Error bars represent SEM (*n* = 3). ns, no significant differences; ** *p* < 0.01, *** *p* < 0.001; ^###^ *p* < 0.001.

To reveal whether Compound A5 has abilities to promote neurogenesis, we examined Compound A5 in primary cortical NSPCs. Indeed, Compound A5 treatment for 5 days has been shown to be capable of enhancing NSPC differentiation, indicated by higher percentages of cells positive for the astrocyte marker GFAP (Figure 2A) and neuronal marker β-tubulin III (Figure 2B). Furthermore, the expression levels of β-tubulin III and GFAP proteins were determined by western blot. Consistently, the up-regulated protein levels for β-tubulin III and GFAP were found (Figure 2C). These results confirmed that Compound A5 enhances neurogenesis in NSPCs.

### 2.3. Compound A5 Promotes Morphological Maturation of Newborn Neurons Derived from NSPCs

**Figure 3:**
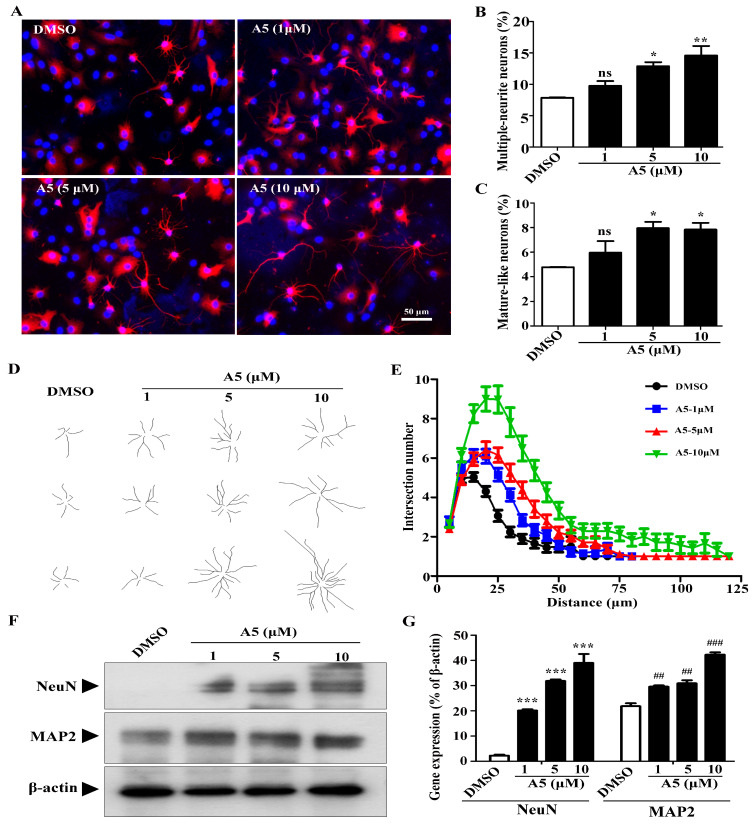
Compound A5 promotes the maturation of newborn neurons derived from NSPCs. (**A**) NSPCs were differentiated for 5 days in the presence of DMSO or different concentrations of Compound A5. Immunostaining of newborn neurons for β-tubulin III (red) and nuclei with DAPI (blue). Scale bar, 50 μm. The percentage of neurons with multiple-neurite neurons (**B**) and Mature-like neurons (**C**) were measured. (**D**) Compound A5 influenced the morphology of mature-like neurons. (**E**) Numbers of dendritic intersections at 0–200 μm from the cell bodies were accessed by *Sholl* analysis. (**F**,**G**) The expression of NeuN and MAP2 was analyzed by Western blot analysis. One-way ANOVA followed by Tukey’s test. Error bars represent SEM (*n* = 3). ns, no significant differences; * *p* < 0.05, ** *p* < 0.01, *** *p* < 0.001; ^##^ *p* < 0.01, ^###^ *p* < 0.001.

During neuron development, cell morphology changes dramatically, the neurites become more extensive, and the branch number of each neuron increases [16]. To gain insights into the effects of Compound A5 during neuron maturation, the number of multiple-neurite neurons was measured for β-tubulin III positive cells (Figure 3A). With Compound A5 treatment, the percentage of multiple neurite neurons (more than two branches) was significantly increased from 7.84 ± 0.05% (DMSO) to 9.75 ± 0.76% (A5, 1 μM) and 12.87 ± 0.65% (A5, 5 μM), and 14.58 ± 1.53% (A5, 10 μM), respectively (Figure 3B). Mature-like neurons had long and extensive dendrites (Figure 3C). Notably, we found that Compound A5 treatment significantly increased the percentage of mature-like neurons from 4.78 ± 0.01% (DMSO) to 5.96 ± 0.95% (A5, 1 μM) and 7.95 ± 0.52% (A5, 5 μM), and 7.84 ± 0.55% (A5, 10 μM), respectively (Figure 3C). Furthermore, the effect of Compound A5 on dendritic complexity was assessed by *Sholl* analysis (Figure 3D,E). Results revealed that Compound A5 could help newborn neurons form a more complicated neurite structure. In accordance with the findings obtained from immunofluorescence staining, the A5-treated cells exhibited an upregulation in the expression of the neuronal differentiation markers MAP2 and NeuN (Figure 3F,G). Above all, we conclude that Compound A5 treatment promotes neurogenesis of NSPCs and results in greater morphological maturity.

### 2.4. Global Analyses of Transcriptome Changes after Treatment with Compound A5 in NSPCs

**Figure 4:**
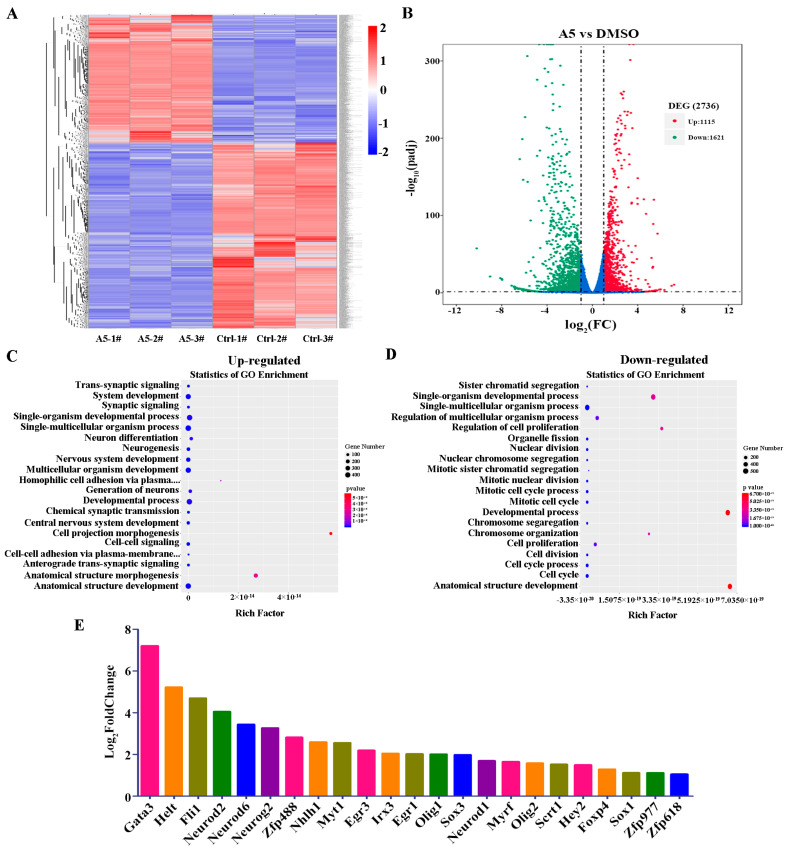
Global analyses of transcriptome changes after treatment with Compound A5 in NSPCs. (**A**) Heatmap showing the hierarchical clustering of DEGs. The horizontal axis represents the sample group names, and the vertical axis shows the normalized values of different genes FPKM. Blue indicates low expression levels, and red indicates high expression levels. (**B**) Volcano plots of differentially expressed genes between Compound A5 and control NSPCs. The results of GO enrichment analysis showed up-regulated genes (**C**) and down-regulated genes (**D**) enriched in the biological process GO term. (**E**) Changes of gene expression in the transcription factors of positive regulation of neurogenesis.

We sought to investigate the molecular mechanism by which Compound A5 promotes neurogenesis in NSPCs, and RNA-seq was performed. Overall, we obtained 2736 genes as differentially expressed genes (DEGs) after treatment with Compound A5, of which 1115 genes were up-regulated, while 1621 genes were down-regulated (Figure 4A,B). To further characterize the potential biological process altered by Compound A5, we performed a Gene Ontology (GO) enrichment analysis. It is worth noting that most DEGs in up-regulated biological processes were related to neural differentiation, such as neuron differentiation, neurogenesis, nervous system development, generation of neurons, and central nervous system development (Figure 4C). Meanwhile, it is also noteworthy that most DEGs in the down-regulated biological process were related to the cell cycle, such as sister chromatid segregation, regulation of cell proliferation, organelle fission, nuclear division, nuclear chromosome, mitotic sister chromatid segregation, mitotic nuclear division, mitotic cell cycle process, mitotic cell cycle, chromosome segregation, chromosome organization, cell proliferation, cell division, cell cycle process, cell cycle (Figure 4D). These results suggested that Compound A5 may promote neurogenesis by up-regulating neurogenesis-related genes and inhibiting cell-cycle progression in NSPCs.

### 2.5. Compound A5 up-Regulates Genes Related to Neurogenesis

According to gene expression analysis, Compound A5 triggers a cascade of biological responses that promote the generation and maturation of new neurons. To further understand the molecular mechanism of Compound A5’s regulation of neurogenesis, we analyzed the transcription factors (TFs) among DEGs induced by Compound A5 in NPSCs. A total of 179 TFs were differentially expressed (80 up-regulated and 99 down-regulated). It is worth noting that the expression of 23 TFs related to neurogenesis was up-regulated, including Gata3, Helt, Flil, Neurod2, Neurod6, Neurog2, Zfp488, Nhlh1, Myt1, Egr3, Irx3, Egr1, Olig1, Neurod1, Myrf, Olig2, Scrt1, Hey2, Foxp4, Sox1, Zfp977, Zfp618, and Sox3 (Figure 4E). These findings provide crucial insights into the molecular mechanisms underlying Compound A5’s role in neurogenesis. Future studies will aim to further elucidate the functions of these up-regulated TFs and their interactions in the neurogenesis process.

### 2.6. Compound A5 Inhibits Cell Cycle Progression in NPSCs

**Figure 5:**
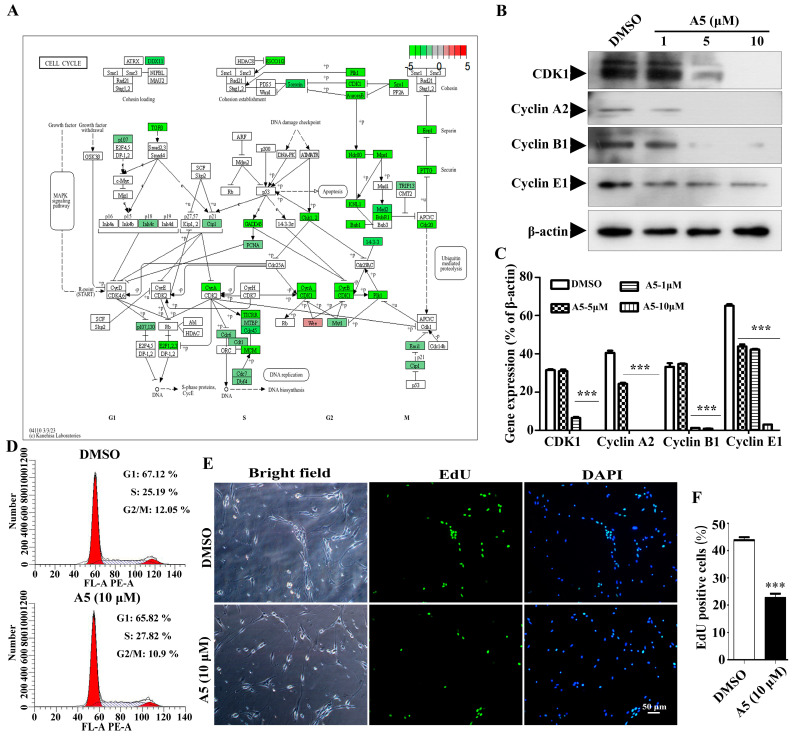
The effect of Compound A5 on cell cycle progression in NPSCs. (**A**). KEGG analysis of cell cycle network. Boxes colored from green to red indicate the magnitude of gene expression. (**B**,**C**) The four genes (CDK1, Cyclin A2, Cyclin B1, Cyclin E1) related to cell cycle regulation were confirmed by Western blotting. One-way ANOVA followed by Dunnett’s test. Error bars represent SEM (*n* = 3). *** *p* < 0.001 compared to the DMSO. (**D**). Flow cytometry was employed to analyze the cell cycle, and the percentages of cell cycle phases were displayed in bar charts. (**E**,**F**). Cell proliferation was assessed using an EdU incorporation assay. *** *p* < 0.001. Student’s *t*-test.

The proliferation and differentiation of stem cells need to be tightly coordinated with the mitotic cell cycle, so the regulation of the cell cycle plays a crucial role in maintaining the stemness and differentiation of stem cells [17,18]. In this study, we found that the down-regulated DEGs exhibited a notable enrichment in the cell cycle process. Subsequently, we mapped the expression status of DEGs across the various stages of the cell cycle as an example (Figure 5A). The cell cycle is a tightly orchestrated sequence of events that leads to cell division and replication. It consists of four distinct phases: G1, S, G2, and M. Each phase is characterized by specific biochemical changes and the activation of specific genes [19]. Compound A5 targets specific key genes (CDK1, CyclinA2, CyclinB1, Cyclin E1) that regulate distinct stages of the cell cycle, effectively blocking the progression of NPSCs from one phase to the next. Additionally, Western blot was used to verify that CDK1, CyclinA2, CyclinB1, and Cyclin E1 are down-regulated in cell cycle regulation (Figure 5B,C). In addition, the effect of A5 on the cell cycle was investigated by flow cytometry. The results showed that the proportion of G1 and G2/M phase cells decreased under the A5 treatment compared with the control, while the proportion of S phase cells increased. This indicated that A5 may induce cell cycle arrest at the S phase (Figure 5D). Moreover, we performed an EdU assay to detect the proliferation of NPSCs treated with Compound A5. We observed that the percentage of cells within neurospheres that incorporated EdU was approximately 43.79 ± 1.1% in the control group, compared to 22.61 ± 1.6% after Compound A5 treatment (Figure 5E,F). The results indicate that Compound A5 can enhance the differentiation of NPSCs into functional neural cells by reducing the rate of cell division.

## 3. Materials and Methods

### 3.1. Reagents

Reagents used in this study were as follows: Compound A5 was synthesized as previously described. Retinoic acid (RA), poly-d-lysine, Laminin, DMSO, MTT, and β-tubulin III antibody (Sigma, St. Louis, MO, USA); GFAP antibody (Santa Cruz, CA, USA); Dulbecco’s modified Eagle’s medium (DMEM/F12) medium, Minimum Eagle’s Medium (MEM), Foetal bovine serum (FBS), Penicillin, and Streptomycin (Hyclone, Logan, UT, USA); B27 and N2 Supplement (Gibco, Grand Island, NY, USA); DAPI, MAP2 antibody, NeuN antibody, CDK1 antibody, Cyclin A2 antibody, Cyclin B1 antibody, Cyclin E1 antibody, β-actin antibody, HRP-conjugated rabbit and mouse secondary antibodies (Beyotime, Shanghai, China); Basic fibroblast growth factor (bFGF) and Epidermal growth factor (EGF) (Peprotech, London, UK); Alexa Fluor-546 goat anti-mouse IgG or Alexa Fluor-488 goat anti-rabbit IgG (Invitrogen, Carlsbad, CA, USA).

### 3.2. Cell Culture

Mouse neuroblastoma cell line Neuro-2a was obtained from the American Type Culture Collection (ATCC, Rockville, MD, USA). Neuro-2a cells were grown in MEM medium supplemented with 10% heat-inactivated fetal bovine serum and 1% penicillin/streptomycin and maintained at 37 °C in a 5% CO_2_ humidified atmosphere. When cells reached 80–90% confluence, cells were passaged by trypsinization. For differentiation, Neuro-2a cells were plated at a density of 2 × 10^4^ cells per well into a 6-well plate, incubated in MEM supplemented with 0.5% FBS for 2 days.

Primary NSPCs were cultured as previously described [20]. For NSPCs differentiation, single cells dissociated from neurospheres were seeded on poly-d-lysine (100 ng/mL) and laminin (20 μg/mL) coated coverslips at 2 × 10^4^ cells/mL. Cells were incubated in DMEM/F12 medium supplemented with 10% FBS and 1% penicillin–streptomycin for 5 days to allow differentiation into multiple lineages in the population.

### 3.3. MTT Analysis

Cell viability was assessed by MTT (3,4,5-dimethylthiazol-2-yl)-2-5-diphenyltetrazolium bromide) reduction assay. For the assay, cells (5 × 10^3^) were plated in 96-well microtiter plates and grown for 24 h. Afterward, cells were treated with different concentrations of Compound A5 (0.1, 0.5, 1, 5, 10 and 20 µM). After 24 h incubation, the media containing Compound A5 were carefully removed, and 100 µL of MTT solution (0.5 mg/mL in MEM) was added to each well and further incubated for four hours. 200 µL DMSO was added to each well to dissolve the formazon crystals, and the absorbance was measured by a microplate reader at 570 nm. Cell viability was shown relative to the control in a graph.

### 3.4. Western Blotting

Cells were lysed in RIPA buffer containing protease and phosphatase inhibitors, and whole cell lysates were quantified using a BCA protein assay kit according to the manufacturer’s instructions. Subsequently, those cell lysates were separated by SDS-PAGE and transferred to PVDF membranes. The membranes were then probed with primary antibodies, followed by incubation with secondary antibodies, and finally detected by electrochemiluminescence (ECL).

### 3.5. Immunostaining

For immunostaining, cells were fixed in freshly prepared 4% PFA for 20 min and then permeabilized in PBS with 0.4% Triton X-100. Following blocked in PBS with 5% goat serum and 1% bovine serum albumin (BSA) for 20 min. The cells were incubated at 4 ℃ overnight with primary antibody, followed by incubation with Alexa Fluor-546 goat anti-mouse IgG or Alexa Fluor-488 goat anti-rabbit IgG as secondary antibodies for 1 h at room temperature. DAPI was added to visualize the nuclei. Images were taken using a fluorescence microscope (Olympus IX71, Tokyo, Japan).

### 3.6. Differential Expression Analysis by RNA-Seq

NSPCs were harvested after treatment with 10 μM A5 or with DMSO for 48 h, and each treatment had three duplicates. Total RNA was extracted using TRIzol reagent (Life Technologies, Waltham, MA, USA). Transcriptome sequencing was performed by Novogene Biotechnology Co., Ltd. (Beijing, China) based on the Illumina Hiseq platform. Gene expression levels were determined by cufflinks in the form of fragments per kilobase of exon per million fragments mapped (FPKM). Differentially expressed genes were determined using the edgeR package; we calculated the log_2_ fold change value (log_2_FC) for each gene in treatment and control samples. Functional enrichment analysis including Gene Ontology and KEGG pathways, was performed for the identified differentially expressed genes.

### 3.7. Cell Cycle Assay by Flow Cytometry

NSPCs were trypsinized and harvested at 350× *g* for 5 min at 4 ℃. Subsequently, cells were fixed in ice-cold 70% ethanol overnight at −80 ℃. Fixed cells were washed with PBS, then digested with stained with RNase A (100 μg/mL) and stained with propidium iodide (50 μg/mL). The stained NSPCs were analyzed with a flow cytometer (Beckman Coulter Inc., Chaska, MN, USA).

### 3.8. EdU Assay

The effect of Compound A5 on the proliferation of NSPCs was determined by the EdU cell proliferation image kit (Abbkine, Wuhan, China), according to the manufacturer’s instructions. The images were taken under a fluorescent microscope (Olympus IX71, Tokyo, Japan). Finally, the percentages of EdU positive cells were counted and analyzed by Image J Version 1.8.0 (https://imagej.net).

### 3.9. Statistical Analysis

The results are expressed as the mean ± standard error of the mean (SEM). The normality of the sample distribution was assessed using the Shapiro–Wilk test. The data were subjected to a Student’s *t*-test or one-way analysis of variance (ANOVA) followed by Tukey’s test to assess the differences between the relevant control and each experimental group. A value of *p* < 0.05 was considered statistically significant.

## 4. Conclusions

In this study, we identified Compound A5, a neuritogenic agent previously designed and synthesized by our group, as a potent inducer of neurogenesis in NSPCs. To further elucidate the molecular mechanisms underlying Compound A5’s neurogenesis-promoting effects, we employed high-throughput Illumina RNA-seq technology for transcriptome analysis. Notably, our comparative transcriptome analysis revealed that the up-regulated genes were primarily associated with neurogenesis, while the down-regulated genes were primarily concerned with cell cycle progression. This observation provides further insights into the compound’s mechanism of action and suggests that it may exert its effects through the modulation of these key regulatory genes.

Collectively, our findings identify Compound A5 as a novel neurogenic agent with the potential to promote neural regeneration. This compound offers a promising starting point for further drug discovery efforts aimed at developing therapeutics for neurodegenerative diseases and brain injuries. Future studies will likely focus on optimizing the compound’s bioactivity and exploring its therapeutic potential in preclinical models of neural injury and disease.

## Data Availability

The original contributions presented in the study are included in the article, further inquiries can be directed to the corresponding author/s.
